# Supplementary material for: Toward a scalable framework for reproducible processing of volumetric, nanoscale neuroimaging datasets
Source: Gigascience. 2020 Dec 21;9(12):giaa147. doi: 10.1093/gigascience/giaa147 (PMC7751400; doi:10.1093/gigascience/giaa147)
Supplement: giaa147_Response_to_Reviewer_Comments_Original_Submission [file giaa147_response_to_reviewer_comments_original_submission.pdf]

Author's Response To Reviewer Comments

Close

Dear Gigascience Editors,

We would like to thank the reviewers and editor for their thoughtful and thorough review of our manuscript, and we are happy to present our revisions to the manuscript. Below, we attempt to highlight how these revisions respond to specific concerns from the reviewers, and improve the quality of the submission accordingly. We have also submitted our tool "Scalable Analytics for Brain Exploration Research" to scicrunch.org, with the resulting RRID:SCR_018812, which has been added to the manuscript. We look forward to hearing your feedback on this improved manuscript. We apologize for the delay in providing this response and appreciate your understanding.

Thank you very much,

Erik C. Johnson, on behalf of all authors

Detailed Response:

Reviewer 1 raised some concerns about the manuscript. We highlight these, along with our response and changes. In addition to the responses below, we made several minor modifications, made capitalization consistent, and changed the color scheme of the table in response to Reviewer 1's comments.

Comment: "I think the reproducibility aspect relates to the processing, not to the reproducibility of the framework. The generality of the term "neuroimaging" had me anticipate a truly generic solution. However, the manuscript itself is focused on a specific subset of data modalities, with specific flavors of being "large". It is unclear to me, if the proposed solution is capable and/or meaningful to be applied to other neuroimaging data, such as MRI, PET, MEG, etc. It would be good to clarify that directly in the title/abstract."

Response: We agree with the reviewer and do not wish to mislead the readers as to the applicability of our software framework. We propose modifying the title to
"Toward A Scalable Framework for Reproducible Processing of Volumetric, Microscale and Nanoscale Neuroimaging Datasets"
To clarify the neuroimaging modalities that work well in this framework (EM, X-ray Microtomography, and Light microscopy) that are best suited for this framework. This is also discussed in a new paragraph in the discussion section. We additionally note in the discussion that this framework is suitable for large-scale batch processing in many domains, but that our specific use-cases focus as described above.

Require technical Expertise and deployment

Comment: "The paper poses the lack of computational expertise and resources as a major challenge regarding large-scale data analysis. I personally support this claim. However, it is not clear to me, if the proposed solution can successfully solve this problem in its entirety, or to which degree. In some sense, the proposed solution is assembled from a substantial number of extremely capable, but also rather complex technologies. Therein lies both potential and difficulty regarding the "lack of expertise". The manuscript, in its present form, does not convince me that SABER/CONDUIT can indeed _lower_ the technical threshold, as opposed to presenting a different set of technical challenges when compared to other solutions. It would be instrumental to know what exactly the path to adoption for a research group (would) look like."

Response: The reviewer has made a great point. We have not conducted extensive usability studies to report here. Anecdotally, however, we believe this is some merit to this claim, particularly in the Electron Microscopy community. This community has repeatedly struggled due to tools which are difficult to install, use and compare. Users need to learn unrelated and often incompatible software libraries and

interfaces. While the SABER framework does not address all issues reducing adoption of new tools, we do believe we can contribute in addressing standardization and compatibility issues. This space is severely underpopulated in terms of interoperable technologies and solutions.

Specifically, we believe the standardized library described in the section “Standardized Workflows and Tools” does help address the required background skills and experience required to run different tools required by the community. It provides an opportunity and a mechanism for labs and tools to work together.

However, the reviewer’s point is still well taken. We have added a section “Required Background and Getting Started” that addresses the required path for adoption and computational expertise still required to utilize this framework. We hope that future work (now discussed in the section “Potential implications”) will integrate this framework into existing datastores and provide graphical user interface tools to enable users with limited.

Comment: “Is it AWS only? must data be in BossDB? What if these 3rd-party services are discontinued?”

Response: The computation framework is flexible in terms of backend computation, due to the use of the Apache Airflow project, although currently local docker execution and AWS batch are supported. We hope additional resource schedulers will be added as the community requires/demands, and the software is released open source for others to adapt as they wish.

For storage, files can be stored locally (or on any locally mounted drive) in numpy or hdf5 files, stored on AWS S3, or stored in the BossDB or DVID systems. This is clarified in the “Cloud Computation and Storage” section in “Methods” in response to this review.

The dependence on 3rd party solutions (AWS, Airflow, bossDB, Datajoint) is certainly a risk if these technologies fail to be supported and developed successfully. However, we believe the strength of building on these existing tools outweighs this risk. For storage and computation, this risk is somewhat mitigated by a modular approach that allows for different operators and datastores. A short discussion of this limitation has been added to the “Discussion” section.

Comment: “what technical expertise needs to exist in a group that aims to adopt this solution?”

Response: Generally speaking, the group needs experience in python and the use of docker containers. Experience with linux systems is preferable, and an AWS account is currently required for scalable computing (although not required if using local resources). While these are non-trivial, we believe that this experience is becoming more widespread (e.g., CS undergraduate student) and we are continuing to lower the barrier to entry through GUI development and improved training tools.
In response to this review, a new section “Required Background and Getting Started” has been added to address this issue as it currently stands in this manuscript.

Comment: “what deployments already exist? Or in other words: what empirical evidence exists that intellectual merits of the proposal translate into practical advantages?”

Response: The SABER platform is an emerging research tool, and this manuscript will help us to socialize the tools with the broader community. We do actively develop and share workflows using this platform for many of our heterogenous research products and especially with our collaborators at Georgia Tech. We have enabled generation of new data products such as synapse labels, segmentations, and cell density analysis as well as benchmarking of algorithms in the projects listed below. We are working on widespread community adoption, but have not reached that point yet, which is a fair criticism - and hopefully enabled by this manuscript.

We are integrating our solution with the bossDB access portal (http://bossdb.org/) to process the datasets hosted there to help create an ecosystem for the community. Our collaborators at Georgia Tech are using these techniques for processing X-ray microtomography data. The underlying tools that we leverage in our platform are used in many applications.

Example uses of the tools so far include:
- Unsupervised learning pipelines for EM processing (https://ieeexplore.ieee.org/abstract/document/9048673)
- Processing pipelines for new xray microtomography datasets

(https://www.biorxiv.org/content/10.1101/2020.05.22.111617v1.abstract)
● Benchmarking novel optimization approaches for workflows (https://arxiv.org/abs/2006.02624)
● NIH Brain Initiative Symposium, poster, and demonstrations (Symposium 5)
https://www.labroots.com/ms/virtual-event/2020-6th-annual-brain-initiative-investigators-virtual-meeting/agenda

Details, open source development and interfacing with other systems:

Comment: The manuscript contrasts SABER/CONDUIT with other tools like LONI and Nipype, and advertises that it frees researchers from being locked into specific idiosyncrasies of these tools and their limitations (need a cluster/no cloud, etc.), and describes an "ecosystem of neuroimaging data analysis pipelines". However, it is unclear from the manuscript in how SABER/CONDUIT does this better than the rest.

Response: While existing pipeline tools like LONI and Nipype enable the execution of scientific workflows, they are lacking a few key features for the neuroimaging user.

First is the library of tools required for modern segmentation and detection problems on EM data, including GPU enabled DNN tools. Second is the orchestration of docker containers, which enable tools with conflicting software dependencies and operating systems. Third are tools to deploy this easily over a single large dataset (parameterize jobs over a single large dataset, optimize multistage workflows end to end). We believe these key features will encourage adoption compared to existing tools.

We have attempted to address this comment by adding additional clarification to the caption of Table 1, as well as to the final two paragraphs of “Existing Software Solutions” in the “Methods” section.

Comment: “ how open is that ecosystem? What is needed to contribute to it? The repository at https://github.com/aplbrain/saber only shows two dozen commits, most done very recently, and not a single pull request. Is this the place where core development is taking place?”

Response: The reviewer raises a great question given the relative newness of the project. The project was released open source just prior to submission of the manuscript. Contributors are now openly developing, creating pull requests, and issues addressed by the team. While the list of contributors is small, we are growing the team. This is now the current repo for core development. We hope momentum will continue to build over time as more users and contributors join the project.

Comment: “which aspects/components of the proposed solution are generic vs specific to the demo'ed data analyses and modalities?”

Response: The reviewer is right that to ensure broad impact, the system needs to be as general as possible. The tools and pipelines are developed for volumetric datasets like EM and XRM; although they perform repeated (e.g., batch) generic problems like object detection, classification, and 2D and 3D segmentation, the parameters and weights provided are specific to these modalities. Users can adapt them to their own problem using the provided optimization framework, but may require annotated data.

The backend code for execution of CWL pipelines of docker containers over large datasets is generic and applicable to any processing pipelines applied over volumetric data.
We have attempted to address this concern through additions in the section “Pipelines and Tools for Neuroimaging Data” as well as with a new paragraph in the “Discussion” section.

Comment: why is CWL better than, say, a workflow definition in Python, as done by Nipype? I do believe that it is, and other projects are moving in this direction, but the manuscript does not make much of a point in this regard.

Response: The reviewer raises a great point about the use of CWL compared to other approaches. We believe that the common, interoperable standard is important to 1) allow reuse of the pipelines in other systems 2) allow pipelines developed for other open source project to be deployed using the SABER/CONDUIT system and 3) reduces the need to yet another workflow specification specific to the system, as is typically done in python workflow managers. This approach leverages the work done by the CWL community.
We have added a paragraph discussing the strengths of the CWL approach to the “Discussion” section.

System Design:
Comment: The manuscript left me with a number of open questions regarding the design specifics of the proposed system. I would personally much prefer to have the manuscript parts concerning example analyses (Fig 1, 2, 3, 4, 5) condensed in favor of a more concrete description of the frameworks components (methods, and Fig. 6). How do they interact (APIs, etc)? What motivated the specific choice of these components? Can they be replaced with alternatives, for example, to better fit into existing institutional infrastructure? The ability to interface "abstract data storage" is briefly mentioned, but there are no specifics regarding required properties of such a storage system.

Response: Thank you for this point. To address this comment we have expanded the discussion of the framework and components, and the discussion of Figure 6.
We have tried to address:
1. API definition
2. Design choices for each component
3. Alternative approaches for different components
We have also addressed the comment on abstract data storage. We hope these changes address the reviewer’s concerns. To address these concerns, we have made extensive revisions to the section “Framework Components” on the components, design choices, and APIs.

Comment: Fig. 6 seems to suggest that there is much flexibility in how this system can be made to work, but it remains unclear to me, which components are, e.g., cloud-only. Moreover, I am unsure about the relationship of SABER and CONDUIT (which both seems to live in the same code repository). Fig. 6 depicts it, congruent with the manuscript, as a central system components. However, it is only very briefly described in the methods section. It seems to me that SABER users would actually mostly interact with CONDUIT, while SABER comprises (in addition) the execution and data access infrastructure. What exactly is CONDUIT adding to cwl-airflow?

Response: Thank you for pointing out these confusing issues. The CONDUIT core is the python code and scripts that build upon CWL and airflow. This includes parsing the CWL and deploying DAGs, as in cwl-airflow. Features added on top of this basic functionality include parameterizing jobs for deployment over chunks of data (specified by coordinates), iterative execution of the same DAG with different parameters (for parameter optimization), and logging of metadata and job results. Moreover, wrappers for the use of local files and s3 files for intermediate results with the same workflows. These features are critical for our machine learning pipeline deployment.
SABER is the collection of tools and workflows, including docker containers and python code for data access, processing, and so forth. A user looking to deploy tools to new data would primarily work with CONDUIT, and a tool developer primarily work with SABER (to ensure compatibility with existing tools). These changes can be seen in Figure 6, as well as in the subsection SABER of the section Methods.

Reviewer 2
Reviewer 2 also raised several key points about the manuscript, which we have responded to; these resulted in significant improvements to the manuscript.

Comment: The authors should identify the likely users of SABER/CONDUIT. For example, would these be labs new to neuroimaging analysis or would they be users with experience who are working with already functioning software pipelines and want more modularity? What are the likely barriers to adoption for the community of interest, and how has this software solution been created so as to minimize these barriers?

Response: This is a great point, thanks for this insight. We imagine this would be established neuroimaging labs with some analysis experience. Our envisioned target users are:
1. Neuroimaging labs wanting to apply tools to new collected datasets to segment data, detect objects of interest, and perform analysis
2. Tool developers who want to package tools to reach new users
We have found that neuroimaging labs have a lot of issues with getting new software tools working on their systems, and deploying those tools at scale. Often, this never happens.
Our solution addresses this issue through several features.
1. A set of dockerized tools to replace installing many, often conflicting dependencies with a single tool (docker)
2. Use of standard CWL definitions which are cross-compatible with other efforts
3. Specialized scripts to handle scheduling runs over large datasets using cloud computing resources
The solution attempts to balance the flexibility needed by tool developers with standardization to help

the novice user.
We have added a section “Required Background and Getting Started” to address these issues.

Comment: Cell identification and connectome maps are generated independently from two distinct tools and datasets (X-ray and EM, respectively). Presumably, many users would be interested in integrating the two outcomes, however no explicit connection is drawn or discussed regarding how complementary outcomes can be correlated and analyzed together through this software.

Response: The reviewer raises a good point- this is in fact possible in this framework. The tools and datasets can be combined in several ways using CWL and docker containers within our framework.
1. The same tools can be used for different steps of both workflows. For instance, our U-net tool can be used to generate probability maps for synapses, cell bodies, or cell membranes when training with different data.
2. Co-registered datasets can be jointly analyzed using our CWL pipelines, and results can all be stored in the bossDB system. This allows the user to use simple python scripts to pull and analyze any parts of these data.
We have added language discussing this to the second paragraph of the discussion.

Comment: In general, it would be useful for the software to include example data that are fully analyzed, together with a mechanism for comparison with user datasets. This would help to identify how datasets from different sources vary, for example for determining the impact of data collection methodology and format, as opposed to differences in the biological system under study.

Response: This is a great point. We have provided some previously processed and existing datasets. First, the fully analyzed datasets in the figures are included with gigascience submission for existing figures.
Moreover, many raw data and processed output datasets are hosted at https://bossdb.org/projects/ and accessible using our bossDB access tools. Users can quickly modify workflows to run on their data and pull this reference data from bossDB for comparison.
This should allow users to make these comparisons, combined with our existing metrics code from our workflows. We have added a section “Datasets for Benchmarking Workflows” to address this.

Comment: The section entitled "Optimization and Deployment of Workflows" is vague and generally unclear.

Response: Thank you for the feedback, we’ve attempted to expand the details in this section to better explain the purpose and the solution. We have revised this section to clearly list the features which enable 1) large-scale deployment of the workflows in the proceeding section and 2) fine-tuning of workflow hyperparameters for deployment to new datasets. We have explicitly listed these use cases and the features which enable them, and we hope this clarifies the need for this section.

Comment: The "Existing Software Solutions" section seems more appropriate for the Background section.

Response: Thank you for the organizational suggestion, while this is certainly important background, we preferred to avoid this detailed discussion of specific software features in the introduction, which we saved for the detailed discussions of software architecture and features later in the manuscript. The point, however, is taken and we have expanded the paragraph in the Introduction beginning with “In other domains, computer science solutions exist for improving algorithm portability and reproducibility” to capture the key features of this information and reference the table.

Comment: The Table 1 caption includes extensive discussion that should be in the main text of the document, rather than the caption. There is little discussion of this table in general, although it is quite valuable.

Response: Thank you for this suggestion, we are glad the table provides a useful comparison of features. We expanded the discussion of this table in “Existing Software Solutions” to provide key context in comparing the features of SABER with other pipeline approaches.

Comment: “In Figure 5, it would be good to include the raw image and to color-code the membranes and synapses for clarity, rather than using black and white.”

Response: Thank you for this suggestion for color coding. We used the standard tool neuroglancer (https://github.com/google/neuroglancer) to generate this plot, and wanted to remain consistent with the visualization hosted online, e.g. at bossdb.org. Probability maps in this format are not visualized with colors (synapses and membranes), whereas annotation ids are. We hope this explains the formatting of the images to be consistent with the existing tools, e.g. the visualizations of data hosted at https://bossdb.org/projects/. We have noted this in the figure caption.

Close
