# Supplementary material for: Toward a scalable framework for reproducible processing of volumetric, nanoscale neuroimaging datasets
Source: Gigascience. 2020 Dec 21;9(12):giaa147. doi: 10.1093/gigascience/giaa147 (PMC7751400; doi:10.1093/gigascience/giaa147)
Supplement: giaa147_Response_to_Reviewer_Comments_Revision_1 [file giaa147_response_to_reviewer_comments_revision_1.pdf]

Author's Response To Reviewer Comments

Close

We have made the required changes from the reviewers, and checked to change minor formatting and typo errors as well.

Close
