# Supplementary material for: Toward a scalable framework for reproducible processing of volumetric, nanoscale neuroimaging datasets
Source: Gigascience. 2020 Dec 21;9(12):giaa147. doi: 10.1093/gigascience/giaa147 (PMC7751400; doi:10.1093/gigascience/giaa147)
Supplement: giaa147_Reviewer_1_Report_Original_Submission — Michael Hanke -- 7/19/2019 Reviewed [file giaa147_reviewer_1_report_original_submission.pdf]

Reviewer Report

Title: Toward A Scalable Framework for Reproducible Processing of Volumetric, Nanoscale Neuroimaging Datasets

Version: Original Submission **Date:** 7/19/2019

Reviewer name: Michael Hanke

Reviewer Comments to Author:

First of all, it is great to see the open-source research ecosystem growing to encompass tools for neuroscience-ready large-scale data analysis. I want to thank the authors for their contributions to the community.
Before I am getting into my review, I want to state that I am neither an expert in the data modalities, nor the specific exemplary analysis approaches that are in the focus of this manuscript.
I struggled a bit with the paper, and had to reconceptualize its aims several times. I will provide a list with some key aspects of my struggle below, and I would appreciate if some of those could be addressed prior publication. I will organize them in the order that they initially occurred to me while reading the paper.
1. Title: "toward a reproducible [...] framework for [...] neuroimaging datasets"
I think the reproducibility aspect relates to the processing, not to the reproducibility of the framework.
The generality of the term "neuroimaging" had me anticipate a truly generic solution. However, the manuscript itself is focused on a specific subset of data modalities, with specific flavors of being "large". It is unclear to me, if the proposed solution is capable and/or meaningful to be applied to other neuroimaging data, such as MRI, PET, MEG, etc. It would be good to clarify that directly in the title/abstract.
2. Expertise (and lack thereof)
The paper poses the lack of computational expertise and resources as a major challenge regarding large-scale data analysis. I personally support this claim. However, it is not clear to me, if the proposed solution can successfully solve this problem in its entirety, or to which degree. In some sense, the proposed solution is assembled from a substantial number of extremely capable, but also rather complex technologies. Therein lies both potential and difficulty regarding the "lack of expertise". The manuscript, in its present form, does not convince me that SABER/CONDUIT can indeed _lower_ the technical threshold, as opposed to presenting a different set of technical challenges when compared to other solutions.
It would be instrumental to know what exactly the path to adoption for a research

group for look like. For example (but not exclusively):
- is it AWS only? must data be in BossDB? What if these 3rd-party services are discontinued?
- what technical expertise needs to exist in a group that aims to adopt this solution?
- what deployments already exist? Or in other words: what empirical evidence exists that intellectual merits of the proposal translate into practical advantages?

3. Interfacing with other systems, and openness of the ecosystem
The manuscript contrasts SABER/CONDUIT with other tools like LONI and Nipype, and advertises that it frees researchers from being locked into specific idiosyncrasies of these tools and their limitations (need a cluster/no cloud, etc.), and describes an "ecosystem of neuroimaging data analysis pipelines". However, it is unclear from the manuscript inhowfar SABER/CONDUIT does this better than the rest. It would be good to give clear pointers on
- how open is that ecosystem? What is needed to contribute to it? The repository at https://github.com/aplbrain/saber only shows two dozen commits, most done very recently, and not a single pull request. Is this the place where core development is taking place?
- which aspects/components of the proposed solution are generic vs specific to the demo'ed data analyses and modalities?
- why is CWL better than, say, a workflow definition in Python, as done by Nipype? I do believe that it is, and other projects are moving in this direction, but the manuscript does not make much of a point in this regard.

4. System Design
The manuscript left me with a number of open questions regarding the design specifics of the proposed system. I would personally much prefer to have the manuscript parts concerning example analyses (Fig 1, 2, 3, 4, 5) condensed in favor of a more concrete description of the frameworks components (methods, and Fig. 6). How do they interact (APIs, etc)? What motivated the specific choice of these components? Can they be replaced with alternatives, for example, to better fit into existing institutional infrastructure? The ability to interface "abstract data storage" is briefly mentioned, but there are no specifics regarding required properties of such a storage system.
Fig. 6 seems to suggest that there is much flexibility in how this system can be made to work, but it remains unclear to me, which components are, e.g., cloud-only. Moreover, I am unsure about the relationship of SABER and CONDUIT (which both seems to live in the same code repository). Fig. 6 depicts it, congruent with the manuscript, as a central system components. However, it is only very briefly described in the methods section. It seems to me that SABER users would actually mostly interact with CONDUIT, while SABER comprises (in addition) the execution and data access infrastructure. What exactly is

CONDUIT adding to cwl-airflow?
Minor
The term "Python" is capitalized inconsistently.
Please consider a different color scheme for Table 1 that is more accessible
for readers with color discrimination difficulties.
https://seaborn.pydata.org/tutorial/color_palettes.html#qualitative-color-palettes

Level of Interest

Please indicate how interesting you found the manuscript: Choose an item.

Quality of Written English

Please indicate the quality of language in the manuscript: Choose an item.

Declaration of Competing Interests

Please complete a declaration of competing interests, considering the following questions:

- Have you in the past five years received reimbursements, fees, funding, or salary from an organisation that may in any way gain or lose financially from the publication of this manuscript, either now or in the future?
- Do you hold any stocks or shares in an organisation that may in any way gain or lose financially from the publication of this manuscript, either now or in the future?
- Do you hold or are you currently applying for any patents relating to the content of the manuscript?
- Have you received reimbursements, fees, funding, or salary from an organization that holds or has applied for patents relating to the content of the manuscript?
- Do you have any other financial competing interests?
- Do you have any non-financial competing interests in relation to this paper?

If you can answer no to all of the above, write 'I declare that I have no competing interests' below. If your reply is yes to any, please give details below.

I declare that I have no competing interests

I agree to the open peer review policy of the journal. I understand that my name will be included on my report to the authors and, if the manuscript is accepted for publication, my named report including any attachments I upload will be posted on the website along with the authors' responses. I agree for my report to be made available under an Open Access Creative Commons CC-BY license (http://creativecommons.org/licenses/by/4.0/). I understand that any comments which I do not wish to

be included in my named report can be included as confidential comments to the editors, which will not be published.

Choose an item.

To further support our reviewers, we have joined with Publons, where you can gain additional credit to further highlight your hard work (see: https://publons.com/journal/530/gigascience). On publication of this paper, your review will be automatically added to Publons, you can then choose whether or not to claim your Publons credit. I understand this statement.

Yes Choose an item.
