# Supplementary material for: Toward a scalable framework for reproducible processing of volumetric, nanoscale neuroimaging datasets
Source: Gigascience. 2020 Dec 21;9(12):giaa147. doi: 10.1093/gigascience/giaa147 (PMC7751400; doi:10.1093/gigascience/giaa147)
Supplement: giaa147_Reviewer_2_Report_Original_Submission — Andrew Michael Smith -- 7/30/2019 Reviewed [file giaa147_reviewer_2_report_original_submission.pdf]

Reviewer Report

Title: Toward A Scalable Framework for Reproducible Processing of Volumetric, Nanoscale Neuroimaging Datasets

Version: Original Submission **Date:** 7/30/2019

Reviewer name: Andrew Michael Smith

Reviewer Comments to Author:

Johnson et al. describe a software framework and analytical workflow for use in processing and analysis of diverse types of imaging datasets, focusing on those collected of the brain through 3D electron microscopy and X-ray microtomography. The authors make the case that there is a need for new tools to extract useful information from large scale neuroimaging datasets and provide a modular software package for processing locally or in the cloud. These presumptions are in general true, and this tool set seems to fit needs for labs looking for universal and adaptable analysis pipelines. I have only minor comments to address.

* The authors should identify the likely users of SABER/CONDUIT. For example, would these be labs new to neuroimaging analysis or would they be users with experience who are working with already functioning software pipelines and want more modularity? What are the likely barriers to adoption for the community of interest, and how has this software solution been created so as to minimize these barriers?
* Cell identification and connectome maps are generated independently from two distinct tools and datasets (X-ray and EM, respectively). Presumably, many users would be interested in integrating the two outcomes, however no explicit connection is drawn or discussed regarding how complementary outcomes can be correlated and analyzed together through this software.
* In general, it would be useful for the software to include example data that are fully analyzed, together with a mechanism for comparison with user datasets. This would help to identify how datasets from different sources differ, for example for determining the impact of data collection methodology and format, as opposed to differences in the biological system under study.
* The section entitled "Optimization and Deployment of Workflows" is vague and generally unclear.
* The "Existing Software Solutions" section seems more appropriate for the Background section.
* The Table 1 caption includes extensive discussion that should be in the main text of the document, rather than the caption. There is little discussion of this table in general, although it is quite valuable.
* In Figure 5, it would be good to include the raw image and to color-code the membranes and synapses for clarity, rather than using black and white.

Level of Interest

Please indicate how interesting you found the manuscript: Choose an item.

Quality of Written English

Please indicate the quality of language in the manuscript: Choose an item.

Declaration of Competing Interests

Please complete a declaration of competing interests, considering the following questions:

- Have you in the past five years received reimbursements, fees, funding, or salary from an organisation that may in any way gain or lose financially from the publication of this manuscript, either now or in the future?
- Do you hold any stocks or shares in an organisation that may in any way gain or lose financially from the publication of this manuscript, either now or in the future?
- Do you hold or are you currently applying for any patents relating to the content of the manuscript?
- Have you received reimbursements, fees, funding, or salary from an organization that holds or has applied for patents relating to the content of the manuscript?
- Do you have any other financial competing interests?
- Do you have any non-financial competing interests in relation to this paper?

If you can answer no to all of the above, write 'I declare that I have no competing interests' below. If your reply is yes to any, please give details below.

I declare that I have no competing interests.

I agree to the open peer review policy of the journal. I understand that my name will be included on my report to the authors and, if the manuscript is accepted for publication, my named report including any attachments I upload will be posted on the website along with the authors' responses. I agree for my report to be made available under an Open Access Creative Commons CC-BY license (http://creativecommons.org/licenses/by/4.0/). I understand that any comments which I do not wish to be included in my named report can be included as confidential comments to the editors, which will not be published.

Choose an item.

To further support our reviewers, we have joined with Publons, where you can gain additional credit to further highlight your hard work (see: https://publons.com/journal/530/gigascience). On publication of this paper, your review will be automatically added to Publons, you can then choose whether or not to claim your Publons credit. I understand this statement.

Yes Choose an item.
